# Bazedoxifene Plays a Protective Role against Inflammatory Injury of Endothelial Cells by Targeting CD40

**DOI:** 10.1155/2020/1795853

**Published:** 2020-12-14

**Authors:** Wenmin Song, Yu Lv, Zizhao Tang, Fangqin Nie, Panhao Huang, Qi Pei, Ren Guo

**Affiliations:** ^1^Department of Pharmacy, The Third Xiangya Hospital, Central South University, Changsha, 410013 Hunan, China; ^2^Center of Clinical Pharmacology, The Third Xiangya Hospital, Central South University, Changsha, 410013 Hunan, China

## Abstract

The inflammatory response and oxidative stress play key roles in the formation and development of atherosclerosis. Bazedoxifene is a new IL6/GP130 inhibitor recommended by the FDA for clinical use as a selective estrogen receptor modulator. However, its role in cardiovascular diseases has been poorly studied. In our study, we explored the mechanism of bazedoxifene's protective effect against inflammatory injury of vascular endothelial cells (VECs) stimulated by TNF-*α*. Various methods were used to verify the effect of bazedoxifene on VECs, including a cell viability assay, a wound healing assay, immunofluorescence staining, and western blotting. Our results showed that TNF-*α* could induce inflammatory damage to VECs, which manifested as upregulated expression of CD40, increased production of ROS, enhanced adhesion of THP-1 cells to VECs, and impaired viability and migration of VECs, while bazedoxifene could significantly reduce the endothelial damage caused by TNF-*α*. In addition, we found that an siRNA targeting CD40 dramatically alleviated the VEC damage induced by TNF-*α*. Therefore, we explored the potential relationship between bazedoxifene and CD40. Our data suggest that bazedoxifene has a protective effect against VEC damage induced by TNF-*α* and that its underlying mechanism may be related to the regulation of CD40.

## 1. Introduction

Atherosclerosis (AS) is a chronic inflammatory disease occurring in large and medium arteries and can cause progressive stenosis of diseased vessels. AS in the coronary and carotid arteries can lead to myocardial infarction, cerebral ischemia, and, in severe cases, death [1]. The pathogenesis of AS is extremely complex and not yet well understood. The occurrence of AS involves multiple environmental factors and multiple cell types, including macrophages, vascular smooth muscle cells, and vascular endothelial cells (VECs), and endothelial dysfunction plays a pivotal role in the whole process of AS [2]. Therefore, protecting the intima of arteries and inhibiting the inflammatory injury of VECs play important roles in the prevention and treatment of AS.

CD40, a member of the tumor necrosis factor superfamily, is a type I transmembrane protein receptor that plays an important role in AS [3]. According to recent studies, CD40 is expressed in a variety of cell types, including vascular smooth muscle cells, VECs, platelets, and immune cells (T cells and B cells). CD40 signaling pathways are activated under a variety of pathological conditions and may be associated with inflammation, AS, and thrombosis [4, 5]. CD40L, a ligand of CD40, is a member of the tumor necrosis factor superfamily and was first identified in activated T cells. A large number of studies have shown that the binding of CD40L and CD40 can induce the release of inflammatory factors (IL-1, IL-12, IL-8, TNF-*α*, IFN-*γ*, IL-6, etc.) and promote the expression of a variety of inflammatory genes, including E-selectin, monocyte chemoattractant protein-1 (MCP-1), macrophage inflammatory protein-1 (MIP-1), vascular cell adhesion molecule-1 (VCAM-1), and intercellular adhesion molecule-1 (ICAM-1) [6]. High levels of VCAM-1 and ICAM-1 aggravate inflammatory endothelial damage, promote the rolling of neutrophils and monocytes in the blood vessels, and make them adhere to the damaged endothelium, further promoting the progression of atherosclerotic plaques [7]. Increasing amounts of evidence suggest that CD40 signaling activates multiple downstream pathways, including the NF-*κ*B, MAPK, JAK/STAT, and PI3K/AKT pathways [8]. In addition, CD40L stimulates the production of reactive oxygen species (ROS) in VECs through the PI3K/AKT pathway [9, 10]. ROS are considered to be the driving force of endothelial dysfunction in many pathological conditions [11, 12]. For example, TNF-*α* induces excessive ROS production and subsequent necrotic core formation in vascular lesions, inhibits VEC migration, increases mononuclear tissue factor expression, and activates platelet aggregation [13–16]. Therefore, inhibiting ROS production can effectively prevent the development of AS.

Bazedoxifene is a novel IL6/GP130 inhibitor that has been approved by the FDA as a selective estrogen receptor modulator for the treatment of osteoporosis in postmenopausal women [17]. Recent research by Lin et al. demonstrated that bazedoxifene inhibits the interaction between IL6 and GP130 through competitive binding with the D1 region of GP130, leading to the inactivation of the IL6/STAT3/AKT/ERK signaling pathway, and thus plays an antitumor role in pancreatic cancer [17, 18]. It is obvious that there is strong crosstalk between the CD40/CD40L system and IL6/GP130 in the downstream pathway. Some studies have reported that bazedoxifene may have a protective effect on the cardiovascular system [19]. Bazedoxifene modulates superoxide production and MCP-1 expression induced by AGE-RAGE in VECs [20]. Postmenopausal monkeys treated with bazedoxifene did not experience atherosclerosis progression or related adverse events [21]. Bazedoxifene also alleviates traumatic brain injury by inhibiting MAPK/NF-*κ*B signaling [22]. However, the study of the role of bazedoxifene in vascular biology and diseases is limited, and the mechanism by which bazedoxifene exerts its cardiovascular effects, particularly the extent to which the regulatory effect of bazedoxifene on CD40 levels alters ROS production to play a role in endothelial dysfunction, remains unclear. In this study, we intend to investigate whether the impairment of endothelial function can be reduced by bazedoxifene treatment and report for the first time that bazedoxifene inhibits TNF-*α*-induced endothelial inflammation by targeting CD40, indicating that bazedoxifene may be a new therapeutic drug for AS.

## 2. Materials and Methods

### 2.1. Materials

Anti-CD40 (#28158-1-AP, 1 : 1000), anti-AKT (#10176-2-AP, 1 : 1000), anti-GAPDH (#10494-1-AP, 1 : 10000), and anti-ERK (#16443-1-AP, 1 : 1000) antibodies were purchased from Proteintech (Hubei, Wuhan, China). Anti-AKT-phosphor-Ser473 (#4060, 1 : 1000) and NF-*κ*B p65 (#8242, 1 : 1000) antibodies were obtained from Cell Signaling Technology (Massachusetts, USA). Anti-phosphor-ERK (#461212082, 1 : 1000) was bought from Sigma (St. Louis, MO, USA). Anti-phosphor-STAT3-Tyr705 (#abs130918, 1 : 1000) was bought from absin (China). And anti-STAT3 (#GB11176, 1 : 1000) and anti-ICAM-1 (#GB11106, 1 : 1000) were obtained from Servicebio (Wuhan, China).

### 2.2. Cell Culture

Human umbilical VECs were purchased from Central South University. VECs were cultured in DMEM (high-glucose, Gibco, New York, USA) supplemented with 10% fetal bovine serum (FBS, Kibbutz Beit Haemek, Israel) and maintained in a relatively humidified atmosphere containing 5% CO_2_ at 37°C. VECs were used to perform experiments from the third passage. VECs were washed with phosphate-buffered saline (PBS) twice when the cell density in a culture flask reached more than 80%. Then, cell contacts were disrupted with 0.25% trypsin, and VECs were subsequently centrifuged after the trypsinization process. The VECs were diluted with complete medium for further experiments.

### 2.3. Cell Viability Assay

The MTT method was used to test cell viability. VECs were cultured in 96-well plates at a density of 1 × 10^5^ cells/mL, and 100 *μ*L of cell suspension was added to each well. VECs were pretreated with bazedoxifene (MedChemExpress, USA) for 30 min, and TNF-*α* (#300-01A, Rocky Hill, NJ, USA) was added to the medium for another 24 h of culture. MTT solution was added to each sample, and the cells were incubated for another 4 h. Subsequently, the blue-violet crystals were dissolved in 150 *μ*L formalin solution. Finally, the samples were measured with a microplate reader at 490 nm.

### 2.4. Monocyte-Endothelial Adhesion Assay

VECs were seeded into 96-well plates for 24 h. After pretreatment of VECs with bazedoxifene for 30 min, TNF-*α* was added to the medium for another 24 h of culture. THP-1 cells were stained with 5 *μ*mol/L BCECF-AM (Beyotime, Jiangsu, China) away from light for 30 min, and then, THP-1 cells were added to the VECs for coculture for another 30 min. Subsequently, the wells were washed with PBS twice to the remove cells that did not adhere to the VECs. Monocyte-endothelial cell adhesion was assessed and quantitatively analyzed by the Operetta High Content Imaging System (PerkinElmer, Massachusetts, USA).

### 2.5. Measurement of ROS Levels

VECs were first plated in a 96-well culture plate for 24 h. Subsequently, they were pretreated with bazedoxifene for 30 min. Then, TNF-*α* was added to the medium, and the cells were cultured for 24 h. Dihydroethidium (Beyotime, Jiangsu, China) is an indicator of ROS permeability, and ROS were labeled with 5 *μ*g/mL dihydroethidium in the dark for 30 min. The generation of ROS was analyzed by the Operetta High Content Imaging System in the Cy3 channel.

### 2.6. Cell Migration Assay

VECs were inoculated into a 6-well cell culture plate. Scratches of the same width were made in the VECs with a pipette tip when the cells were 100% confluent. The VECs were washed with PBS twice. After VECs were pretreated with bazedoxifene for 30 min, TNF-*α* was added to the medium, and the cells were cultured for 24 h. After 24 h, the wound was measured by inverted fluorescence microscopy.

### 2.7. RNA Interference

To silence CD40, VECs seeded in 6-well plates were transiently transfected with a small interfering RNA (siRNA) targeting CD40 or a negative control siRNA (Nc-siRNA) for 48 h. Nc-siRNA or siRNA was diluted with the reagents of the riboFECT™ Transfection Kit according to the manufacturer's instructions. The silencing effect of the siRNA was assessed by western blotting after transfection.

### 2.8. Western Blotting

According to the manufacturer's instructions, total cell lysates were extracted with RIPA lysis buffer containing 0.1% PMSF (Beyotime Biotech, China) and denatured at 100°C for 10 min. Total protein was separated by SDS-polyacrylamide gel electrophoresis (Beyotime Biotech, China) and then transferred onto PVDF membranes at 100 V for 80 min. Subsequently, the membranes were blocked with TBST containing 5% nonfat dry milk at room temperature for 60 min and incubated with the corresponding antibody overnight at 4°C followed by a horseradish peroxidase-conjugated secondary antibody (1 : 10000) for 60 min. Immunoreactive proteins were identified by Clarity™ Western ECL Substrate (Berkeley, California, USA). Densitometric analysis of the band intensity was performed using Image Lab software (Bio-Rad).

### 2.9. Real-Time PCR Analysis

Total RNA was extracted from VECs by using TRIzol reagent (Invitrogen, Carlsbad, CA, USA). Real-Time PCR was performed using the ABI 7300 Real-Time PCR system with the SYBR Green Real-Time PCR Kit (CWBIO, Beijing, China). The relative expression of IL-6 was analyzed by the 2^-*ΔΔ*Ct^ method. The sequences of primers were listed as follows: IL-6 forward 5′-GCAATAACCACCCCTGACCCAA-3′ and reverse 5′-GCTACATTTGCCGAAGAGCC-3′; GAPDH forward 5′-TGACTTCAACAGCGACACCCA-3′ and reverse 5′-CACCCTGTTGCTGTAGC CAAA-3′.

### 2.10. Statistical Analysis

Statistical analysis was performed with SPSS software (version 16.0). The data are shown as the mean ± SD. Unpaired Student's *t*-test was used for direct comparisons between two groups. The differences among the groups were compared using one-way analysis of variance (ANOVA). The least significant difference *t*-test (LSD-*t*) was used for pairwise comparisons after ANOVA. *P* < 0.05 was considered statistically significant.

## 3. Results

### 3.1. Bazedoxifene Ameliorates TNF*α*-Induced Damage to VECs

**Figure 1:**
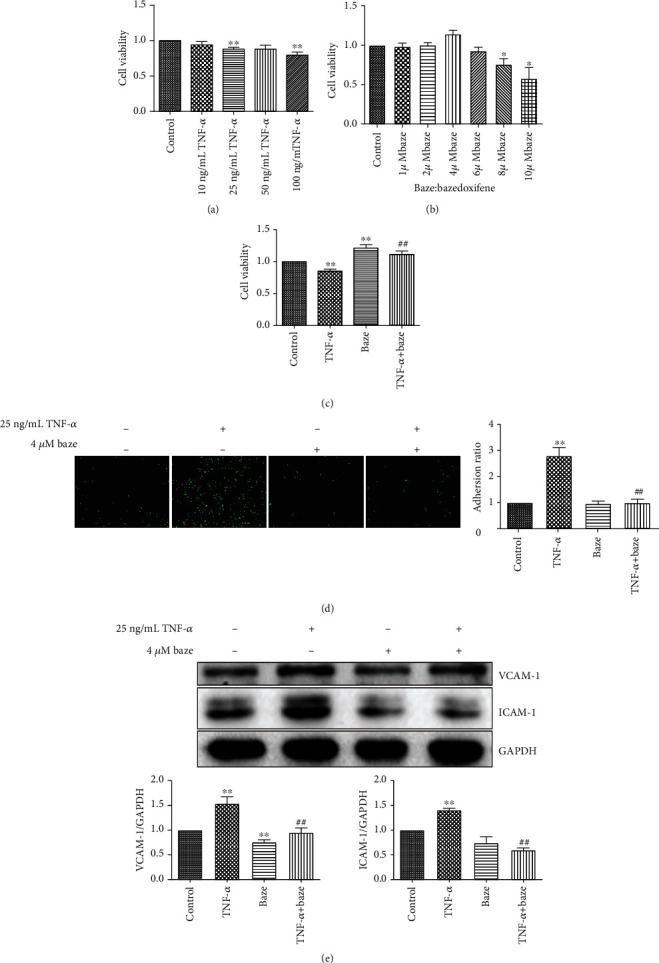
Bazedoxifene plays a protective effect against TNF-*α*-induced VEC inflammation injury and the adhesion between THP-1 and VECs. (a) TNF-*α* decreased VEC viability in a dose-dependent manner when the concentration was higher than 25 ng/mL. (b) Treatment of VECs with different concentration bazedoxifene for 24 h significantly decreased the percentage of viable cells when the concentration was higher than 4 *μ*mol/L. (c) Pretreatment with bazedoxifene at 4 *μ*mol/L prevented TNF-*α*-induced damage on VECs. (d) THP-1 was labeled with BCECF-AM for 30 min and then added to EVCs for coculture for another 30 min. The images were captured by Operetta High Content Imaging System. Scale bar: 200 *μ*m. (e) The expressions of VCAM-1 and ICAM-1. All values are expressed as the mean ± SD (*n* = 3); ^∗^*P* < 0.05 and ^∗∗^*P* < 0.01, compared with the control group; ^##^*P* < 0.01, compared with the TNF-*α* group.

It has been reported that TNF-*α* can be used to establish a stable inflammation model in VECs at a concentration of 10 ng/mL to 50 ng/mL [23–25]. In this study, we administered different concentrations of TNF-*α* (10, 25, 50, and 100 ng/mL) to confirm its effect on VEC activity. As shown in Figure 1(a), 10 ng/mL TNF-*α* had no significant effect on VECs. When the concentration of TNF-*α* was higher than 25 ng/mL, TNF-*α* decreased the activity of VECs in a dose-dependent manner. Therefore, we selected 25 ng/mL TNF-*α* to establish a model of VEC damage and explored the pharmacological effect of bazedoxifene on VECs stimulated by TNF-*α*. In our research, we used the MTT assay to evaluate the cytotoxicity of bazedoxifene. As shown in Figure 1(b), when the concentration of bazedoxifene was higher than 4 *μ*mol/L, bazedoxifene reduced VEC activity in a dose-dependent manner, while low concentrations of bazedoxifene had no significant effect on cell activity. Subsequently, we attempted to confirm whether bazedoxifene can reverse the damage to VECs induced by TNF-*α*. In line with our expectations, 4 *μ*mol/L bazedoxifene reversed the TNF-*α*-induced decrease in VEC viability (Figure 1(c)). These data suggest that bazedoxifene improves the viability of VECs after TNF-*α* stimulation. To test the effect of bazedoxifene on endothelial function, we performed a mononuclear cell adhesion experiment after TNF-*α* treatment. In our study, we labeled THP-1 cells with BCECF-AM and then added them to VECs to establish a coculture system. As shown in Figure 1(d), treatment of VECs with 25 ng/mL TNF-*α* substantially promoted THP-1 adhesion to VECs, and pretreatment of VECs with bazedoxifene attenuated this adhesion, as expected. Previous studies have shown that VCAM-1 and ICAM-1 protein levels are associated with the adhesive and chemotactic abilities of monocytes [26]. Therefore, we measured VCAM-1 and ICAM-1 levels by western blotting to determine the possible pharmacological mechanism by which bazedoxifene affects the interaction between VECs and THP-1 cells. We found that VCAM-1 and ICAM-1 expression was dramatically increased in the TNF-*α*-treated group compared with the control group (Figure 1(e)). However, pretreatment of VECs with bazedoxifene significantly reduced the increase in the expression of VCAM-1 and ICAM-1 induced in the presence of TNF-*α* (Figure 1(e)).

### 3.2. The Effect of Bazedoxifene on TNF-*α*-Induced Migration and ROS Production in VECs

**Figure 2:**
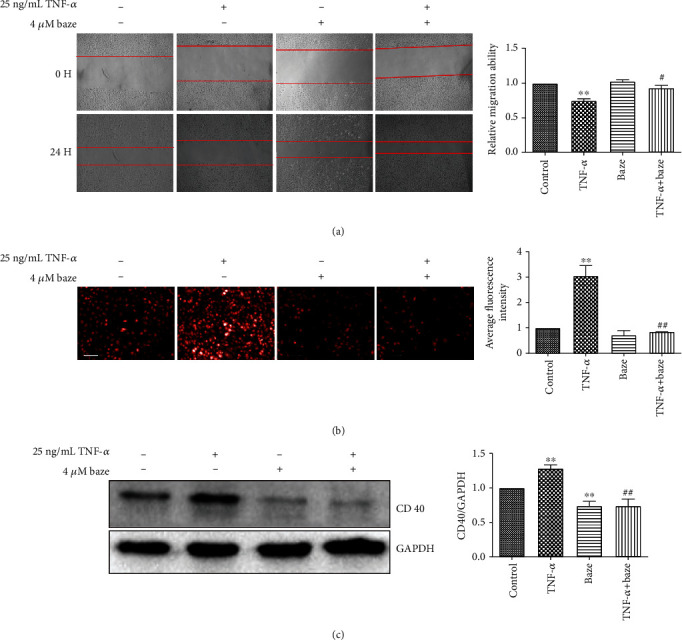
The effect of bazedoxifene on migration and ROS production of VECs induced by 25 ng/mL TNF-*α*. (a) The quantitative result of wound healing. (b) The role of bazedoxifene on the generation of ROS in VECs. VECs were cultured with bazedoxifene with or without TNF-*α* for 24 h. Subsequently, ROS was stained with dihydroethidium at 5 *μ*g/mL for 30 min. And fluorescence intensity was captured and analyzed by Operetta High Content Imaging System. Scale bar: 100 *μ*m. (c) The expression of CD40. The data were expressed as the mean ± SD (*n* = 3); ^∗∗^*P* < 0.01, compared with the control group; ^#^*P* < 0.05 and ^##^*P* < 0.01, compared with the TNF-*α* group.

Studies have shown that elevated levels of TNF-*α* in the surrounding environment can impair VEC function, as indicated by reduced VEC migration [15]. To determine the role of bazedoxifene in VEC migration, VECs were cultured with or without TNF-*α*, and VEC migration was tested by a scratch wound assay. As shown in Figure 2(a), VEC migration was inhibited in the TNF-*α*-treated group compared to the control group, as indicated by the wider space between the separated parts of cells, and bazedoxifene alone had no obvious effect on the migration of VECs. More importantly, pretreatment of VECs with bazedoxifene significantly enhanced endothelial repair upon TNF-*α* treatment (Figure 2(a)). Exposure of VECs to TNF-*α* rapidly increased intracellular ROS levels, as measured by the Operetta High Content Imaging System (Figure 2(b)). Pretreatment of VECs with bazedoxifene significantly attenuated the increase in the levels of ROS in VECs upon TNF-*α* treatment (Figure 2(b)). In addition, VECs stimulated with TNF-*α* showed dramatically increased CD40 expression, and this phenomenon was inhibited by bazedoxifene (Figure 2(c)), indicating that bazedoxifene may exert its effects by targeting CD40.

### 3.3. Bazedoxifene Inhibits JAK/STAT, MAPK/ERK, PI3K/AKT, and NF-*κ*B Signaling Triggered by TNF-*α*

**Figure 3:**
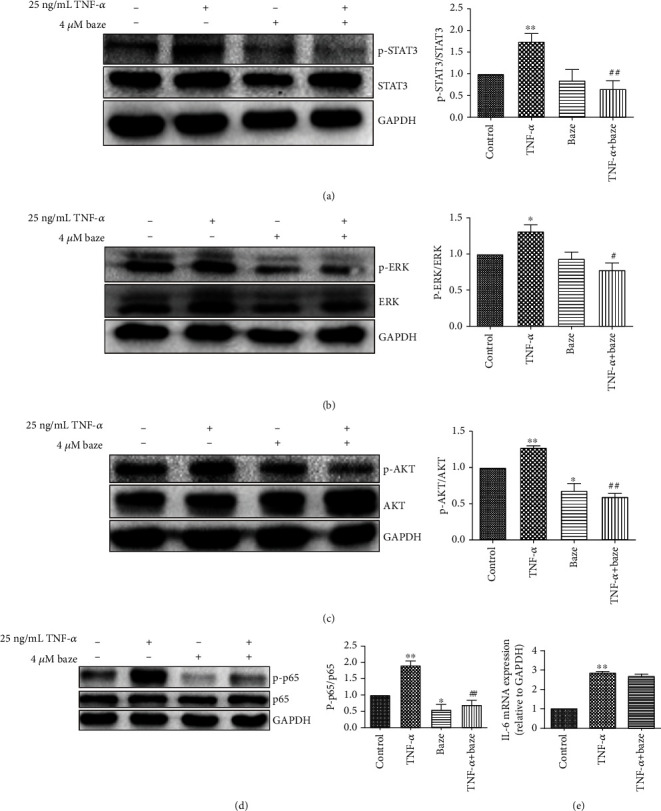
Bazedoxifene protects VECs against TNF-*α*-induced inflammation by multiple pathways. VECs were pretreated with bazedoxifene for 30 min, and TNF-*α* was added in medium for another 24 h treatment. (a, b) The protein levels of p-STAT3 and p-ERK were measured by using western blotting. (c) The protein levels of p-AKT. (d) The nuclear expression of p65. (e) IL-6 mRNA expression in each group. The data were expressed as the mean ± SD (*n* = 3); ^∗^*P* < 0.05 and ^∗∗^*P* < 0.01, compared with the control group; ^#^*P* < 0.05 and ^##^*P* < 0.01, compared with the TNF-*α* group.

The activation of JAK/STAT is essential for the stress response [27]. To investigate whether bazedoxifene has any effect on the TNF-*α*-induced JAK/STAT pathway in VECs, western blot analysis of proteins extracted from VECs subjected to different treatments was performed to evaluate the expression of p-STAT3. As shown in Figure 3(a), the expression of p-STAT3 was significantly increased in the TNF-*α*-treated group compared with the control, and this effect was greatly suppressed by bazedoxifene at a concentration of 4 *μ*mol/L, indicating that bazedoxifene partly inhibited the JAK/STAT3 signaling pathway. In this study, we also attempted to verify the role of the MAPK/ERK pathway in the TNF-*α*-induced VEC damage model and to determine whether bazedoxifene can affect this signaling pathway. As shown in Figure 3(b), p-ERK expression was significantly increased in VECs after TNF-*α* treatment. However, bazedoxifene diminished the upregulation of p-ERK expression induced by TNF-*α*, suggesting that bazedoxifene is an inhibitor of the MAPK/ERK signaling pathway under this condition. In addition, p-AKT expression was stimulated by TNF-*α* (Figure 3(c)), which is consistent with previous reports showing that the PI3K/AKT pathway is involved in ROS production [9]. Pretreatment of VECs with bazedoxifene significantly attenuated the increase in the level of p-AKT in VECs upon TNF-*α* treatment (Figure 3(c)). Our results also showed that bazedoxifene alone could inhibit the expression of p-STAT3, p-ERK, and p-AKT.

To investigate the effect of bazedoxifene on TNF-*α*-induced endothelial inflammation, we also assessed NF-*κ*B activation in this study. As shown in Figure 3(d), TNF-*α* significantly induced the nuclear expression of p65 (p-p65) at the protein level, and bazedoxifene markedly reduced the upregulation of p-p65 expression triggered by TNF-*α*. Our results also showed that bazedoxifene alone could inhibit the expression of p-p65. We also evaluated the IL-6 mRNA level using Real-Time PCR. Interestingly, bazedoxifene did not inhibit the increase in IL-6 mRNA expression induced by TNF-*α* (Figure 3(e)). These data indicate that bazedoxifene plays an anti-inflammatory role independent of the IL-6 signaling pathway.

### 3.4. Bazedoxifene Inhibits TNF-*α*-Induced Inflammatory Injury in VECs by Targeting CD40

**Figure 4:**
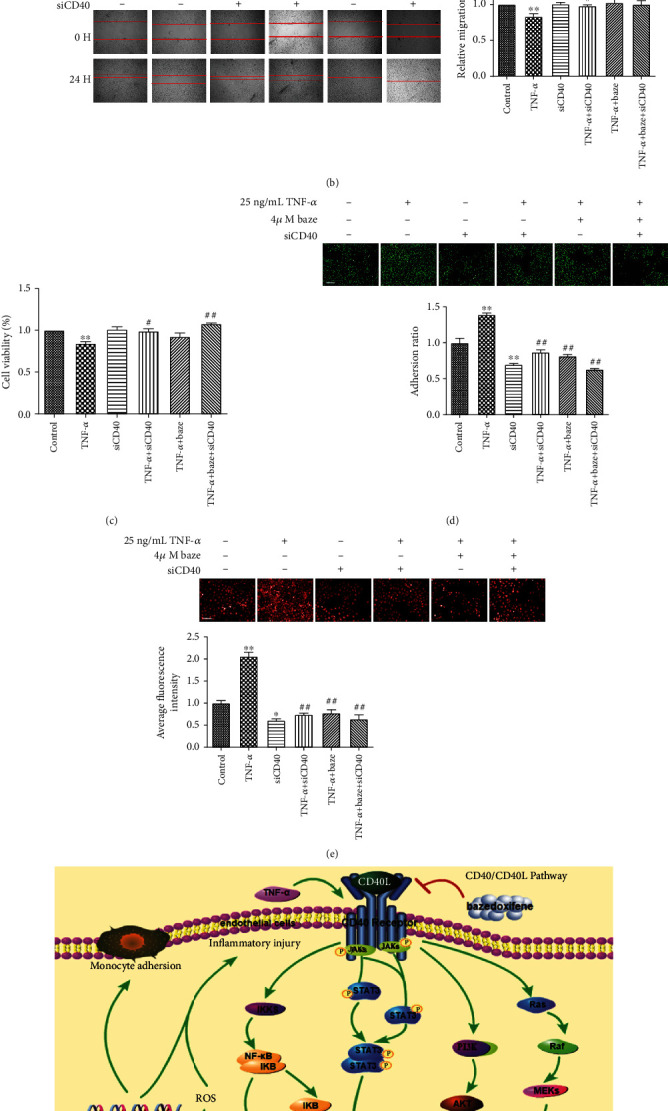
Effect of CD40 on inflammation in VECs. VECs were transfected with siRNA targeting CD40. (a) The CD40 expression after transfection with siRNA targeting CD40. (b) The effect of CD40 on the migration of VECs. (c) The viability of VECs after transfection with siRNA targeting CD40. (d) The adhesion between THP-1 and VECs. Scale bar: 200 *μ*m. (e) ROS production after knocking down CD40. Scale bar: 100 *μ*m. (f) Mechanism, by which bazedoxifene improves TNF-*α*-induced endometrial dysfunction. All values are expressed as the mean ± SD (*n* = 3); ^∗^*P* < 0.05 and ^∗∗^*P* < 0.01, compared with the control group; ^#^*P* < 0.05 and ^##^*P* < 0.01, compared with the TNF-*α* group.

CD40 is a transmembrane protein, and its overexpression can induce the activation of multiple downstream AS-related pathways. Therefore, we further confirmed the role of CD40 in endothelial inflammation by a loss-of-function experiment. As shown in Figure 4(a), CD40 expression was significantly reduced in the siRNA-mediated CD40 knockout group compared to the control and TNF-*α*-treated groups. In addition, the combination of siRNA-CD40 with bazedoxifene further reduced the expression of CD40 compared with single treatment (Figure 4(a)).

In this study, we found that the siRNA targeting CD40 and bazedoxifene were able to reverse the impairment in the migration of VECs induced by TNF-*α*, suggesting that bazedoxifene may play a protective role in VEC function by targeting CD40 (Figure 4(b)). We also found that both CD40 knockdown and bazedoxifene increased VEC viability after TNF-*α* treatment, inhibited monocyte adherence to VECs, and reduced ROS levels after TNF-*α* administration (Figures 4(c)–4(e)). Interestingly, bazedoxifene treatment, however, did not further alter VEC migration, VEC viability, monocyte adherence, and ROS production in CD40 knockdown VECs after TNF-*α* administration, indicating that these effects of bazedoxifene on VECs are CD40-dependent.

## 4. Discussion

A large number of studies have shown that TNF-*α* could establish a stable VEC inflammation model [23–25, 28]. However, the concentration of TNF-*α* used to establish the model may be different by specific cell conditions and techniques. In our research, TNF-*α* at a concentration of 25 ng/mL decreased cell viability to 80% approximately. Therefore, we selected 25 ng/mL TNF-*α* to establish an inflammation model of VECs. Bazedoxifene, a third-generation selective estrogen receptor modulator, is clinically used for the treatment of osteoporosis. Recent studies have confirmed that bazedoxifene is also an effective IL6/GP130 inhibitor [17, 29, 30]. Furthermore, bazedoxifene can inhibit ROS production and MCP-1 levels induced by AGE-RAGE in VECs [20]. However, the cardiovascular effects of bazedoxifene and its role in cardiovascular disease are still poorly understood. In this study, we focused on the protective effect of bazedoxifene against TNF-*α*-induced endothelial dysfunction. According to our data, low-dose bazedoxifene had no significant effect on the viability of VECs, while high-dose bazedoxifene showed some cytotoxicity. Therefore, bazedoxifene at different concentrations was applied to VECs to explore its potential pharmacological effects. Our data indicated that bazedoxifene significantly improved the survival rate of VECs after stimulation with TNF-*α*. Therefore, we believe that bazedoxifene may be an effective drug for the treatment of inflammatory damage to VECs and may protect the endothelium from the damage caused by various inflammatory diseases, such as AS.

CD40/CD40L is a pair of complementary transmembrane proteins that are mainly expressed in a variety of immune and nonimmune cells, including vascular smooth muscle cells, macrophages, lymphocytes, and VECs [31]. Activation of the CD40/CD40L signaling pathway causes the upregulation of the expression of many proinflammatory cytokines and proatherosclerotic genes, which is an essential process in many diseases, particularly AS [3]. The interaction between CD40 and CD40L promotes the adhesion of monocytes to the damaged endothelium, exacerbates local inflammation and oxidative stress levels, further accelerates endothelial damage, and promotes the rupture of unstable plaques in advanced AS [32]. In our study, bazedoxifene dramatically suppressed CD40 expression induced by TNF-*α* in VECs. Given the role of the CD40/CD40L system in the inflammatory response and oxidative stress, bazedoxifene may play an anti-AS role by regulating CD40.

CD40/CD40L may trigger multiple signaling cascades, including the JAK-STAT, PI3K/AKT, and MAPK/ERK pathways, which cause the translocation of the NF-*κ*B p65 subunit from the cytoplasm to the nucleus; upregulate the expression of VCAM-1, ICAM-1, and other proinflammatory genes; promote the infiltration of inflammatory cells; and induce oxidative stress [26, 33–36]. All these pathological changes aggravate local inflammatory injury and aggravate AS [27]. Therefore, preventing the overactivation of the PI3K/AKT, JAK/STAT3, and MAPK/ERK signaling pathways may be a key strategy for anti-AS therapy. Bazedoxifene is a newly identified inhibitor of IL6/GP130 that blocks the phosphorylation of its downstream targets, such as STAT3, AKT, and ERK [37]. The analysis of the respective signal transduction pathways reveals a strong crosstalk between the IL6/GP130 and CD40/CD40L systems. Consistent with recent findings [22, 37], bazedoxifene strongly suppressed TNF-*α*-induced activation of JAK/STAT3, MAPK/ERK, PI3K/AKT, and NF-*κ*B in VECs. More importantly, bazedoxifene protected VECs from TNF-*α*-induced damage, suggesting that bazedoxifene may play a protective role in cardiovascular diseases through regulating the complex network connecting CD40, STAT3, AKT, ERK, and NF-*κ*B signaling.

Increased CD40 and CD40L expression is found in human atherosclerotic plaques, and inhibition of CD40 via gene knockout in mice dramatically reduces the area of atherosclerotic plaques, promotes the stability of plaques, and decreases the risk of plaque rupture [38]. Recently, activation of CD40 was reported to inhibit VEC migration by increasing ROS production [15]. The impairment of VEC migration may seriously affect endothelial regeneration after plaque erosion [32]. Thus, suppressing the abnormal increase in CD40 levels and restoring VEC migration ability can promote plaque stability and prevent thrombosis. Consistent with previous reports, our study showed that CD40 expression was significantly upregulated, ROS production was dramatically enhanced, and VEC migration was significantly impaired in a TNF-*α*-induced VEC damage model. However, these effects were reversed by bazedoxifene. More importantly, bazedoxifene treatment did not further alter VEC migration, VEC viability, monocyte adherence, and ROS production in CD40 knockdown VECs after TNF-*α* administration, suggesting that the effects of bazedoxifene on VEC function are mediated via CD40.

In summary, we showed that bazedoxifene may play an endothelial protective role in a TNF-*α*-induced endothelial damage model through anti-inflammatory and antioxidant pathways. Our work provided evidence that bazedoxifene protects the vascular endothelium by inhibiting the activation of the NF-*κ*B p65, MAPK/ERK, PI3K/AKT, and JAK/STAT3 pathways induced by TNF-*α*. Bazedoxifene attenuates ROS production and the adhesion of monocytes to the damaged endothelium and restores the migration ability of VECs upon TNF-*α* treatment. The inflammation-mediated damaging effect of TNF-*α* on VECs can be abolished by knocking down CD40, indicating that bazedoxifene exerts its protective effect partially through targeting CD40. Our study was the first to investigate whether bazedoxifene's cardiovascular effects could be harnessed to treat endothelial dysfunction and to demonstrate for the first time that bazedoxifene targets CD40 to protect VECs from inflammatory damage. Our study also has extensive implications for the diagnosis and treatment of AS. However, this study also has some limitations. Our data indicate that bazedoxifene plays a role in endothelial protection through CD40, but they cannot explain the pharmacological mechanisms of bazedoxifene.

## Data Availability

The data used to support the findings of this study are included within the article.
